# Multivariate analysis of brain metabolism reveals chemotherapy effects on prefrontal cerebellar system when related to dorsal attention network

**DOI:** 10.1186/2191-219X-3-22

**Published:** 2013-04-04

**Authors:** Federico D'Agata, Tommaso Costa, Paola Caroppo, Bruno Baudino, Franco Cauda, Matteo Manfredi, Giuliano Geminiani, Paolo Mortara, Lorenzo Pinessi, Giancarlo Castellano, Gianni Bisi

**Affiliations:** 1 Department of Neuroscience, AOU San Giovanni Battista, Turin, Italy; 2 Department of Psychology, University of Turin, Turin, Italy; 3 Nuclear Medicine, AOU San Giovanni Battista, Turin, Italy

**Keywords:** Cancer, Chemotherapy, Brain glucose metabolism, Resting state, Support vector machine

## Abstract

**Background:**

Functional brain changes induced by chemotherapy are still not well characterized. We used a novel approach with a multivariate technique to analyze brain resting state [^18^ F]FDG-PET in patients with lymphoma, to explore differences on cerebral metabolic glucose rate between chemotherapy-treated and non-treated patients.

**Methods:**

PET/CT scan was performed on 28 patients, with 14 treated with systemic chemotherapy. We used a support vector machine (SVM) classification, extracting the mean metabolism from the metabolic patterns, or networks, that discriminate the two groups. We calculated the correct classifications of the two groups using the mean metabolic values extracted by the networks.

**Results:**

The SVM classification analysis gave clear-cut patterns that discriminate the two groups. The first, hypometabolic network in chemotherapy patients, included mostly prefrontal cortex and cerebellar areas (central executive network, CEN, and salience network, SN); the second, which is equal between groups, included mostly parietal areas and the frontal eye field (dorsal attention network, DAN). The correct classification membership to chemotherapy or not chemotherapy-treated patients, using only one network, was of 50% to 68%; however, when all the networks were used together, it reached 80%.

**Conclusions:**

The evidenced networks were related to attention and executive functions, with CEN and SN more specialized in shifting, inhibition and monitoring, DAN in orienting attention. Only using DAN as a reference point, indicating the global frontal functioning before chemotherapy, we could better classify the subjects. The emerging concept consists in the importance of the investigation of brain intrinsic networks and their relations in chemotherapy cognitive induced changes.

## Background

Cognitive changes in cancer patients after adjuvant chemotherapy (CHT) have been reported since the mid 1970s, with systematic research starting in the early 1990s. Most neuropsychological studies on CHT-treated cancer survivors have reported cognitive impairments in multiple domains such as executive functions, learning, memory, attention, verbal fluency and speed of information processing. The CHT effects ranges from small to moderate, involving mostly the cognitive functions subserved by frontal lobes [1-3].

PET and magnetic resonance imaging (MRI) brain studies have provided evidences that CHT can induce both structural (white matter [4-8] or grey matter [6,7,9] damages) and functional changes (hypometabolism [10] or hypoactivation [11-13]) in areas correlated with attention, concentration and/or memory (such as the prefrontal, cingulate and parahippocampal cortex). Longitudinal neuropsychological [14,15] and neuroimaging [5,7] studies pointed to a total reversibility after 1 to 3 years, opposed to cross-sectional studies [10,12,16,17] that evidenced, in some patients, a long-lasting damage (5 to 10 years). It is likely that chemotherapy generates a subtle damage, largely reversible for the resolving power of our techniques, but that could constitute, in the long course, a risk factor for the onset of cognitive impairment in some genetically and environmentally vulnerable patients [18,19].

All the cited neuroimaging studies used univariate approaches to identify the presence of focal or local brain changes induced by chemotherapy, trying to find areas in which the null hypothesis could be discarded. In this study, we investigated the chemotherapy effects starting from the brain total activity, instead of comparing two groups of treated and not treated patients for single voxels. We considered the pattern of intrinsic covariation of different brain regions using them to map representative brain characteristics onto the two groups. It is well known that the resting brain contains the temporal overlapping of many spatially independent networks [20], which activity during behavioural tasks is correlated with brain cognitive and emotional functions [21-23]. This is possibly an intrinsic characteristic of the brain architecture, which is a depiction of the need of integration of different areas to substantiate the mind abilities [24].

The cognitive functions that are more fogged by chemotherapy (attention, concentration, memory, speed processing and executive functions) could be suggestive of the networks involved that will be the most representative of these functions [22,25-28] which are the following:

1 The central executive network (CEN), left and right parts, consists mainly of the dorsolateral prefrontal cortex (DLPFC), Brodmann area (BA), 44/45/46, dorsomedial prefrontal cortex, BA 8, 9, inferior and superior parietal lobule, BA 7, supramarginal and angular gyrus, BA 39/40 and cerebellum crus 1 and crus 2.

2 Salience network (SN) consists mainly of the medial frontal cortex, BA 32, the dorsal anterior cingulate cortex, BA 24, anterior prefrontal cortex, BA 10, 11, DLPFC, BA 46, the frontoinsular cortex, BA 47/12, the thalamus and the cerebellum VI and crus 1.

3 Dorsal attentional network (DAN) consists mainly of the superior parietal lobule, BA 5, 7, supramarginal gyrus, BA 40 the frontal eye fields (FEF), BA 6 and cerebellum VI.

We employed the multi-voxel pattern analysis (MVPA), a technique involving searching through data to identify patterns of features that are highly predictive of different conditions. MVPA has been successfully applied in many different medicine fields, for example, in gene selection for large cluster analyses [29] or in detection of ventricular fibrillation [30]. In neuroimaging literature, the use of MVPA for functional MRI analyses (fMRI) [31,32] has become more and more popular after the work of Haxby et al. [33]. The voxels’ activation patterns in the ventral temporal cortex, during a vision task, were distinctive of the category of objects viewed by the experimental subjects (drawing attention to the so-called mind reading field of study [34]). This could be considered a revival in fMRI of the multivariate techniques introduced in PET a decade before [35,36]. In our case, data features were the regional cerebral metabolic glucose rate values, obtained from brain resting state [^18^ F]FDG-PET in lymphoma patients and MVPA extracted spatial patterns, representative of the two different groups brain states (treated and untreated patients).

MVPA, compared to the univariate analysis, could detect subtle distributed effects and might consider the covariance between distinct brain areas as a signature of distributed brain networks. The aim of the study was to uncover the effects of chemotherapy on brain intrinsic networks enclosed in the resting ongoing activity.

## Methods

### Patients

Cancer patients were enrolled among those who were planned to undergo a whole-body [^18^ F]FDG-PET/CT on a clinically routine basis for cancer staging or to monitor the disease after treatment.

Patients were considered eligible if they did not have symptoms of neurological and psychiatric disorders and medications that could potentially alter the neuropsychological performances and/or brain metabolism. We also excluded patients who had, in the previous 2 years, pharmacological or psychological treatments that could potentially alter the brain metabolism or who had serious physical effects after the chemotherapy treatment.

Eligible patients gave written informed consent to participate to the project, approved by the ethical committee of AOU San Giovanni Battista University Hospital in Turin.

Among 28 enrolled patients, 14 were previously treated with systemic CHT and 14 patients were not treated (no CHT). The two subgroups were balanced for age (mean 52 years old), gender (12 males, 2 females) and education level. All selected patients were evaluated and treated in the same Haematology Oncology Department. In both groups, majority of the patients had a non-Hodgkin's lymphoma (NHL) (*n* = 12, 86%), while minority had a Hodgkin’s lymphoma (HL) (*n* = 2, 14%). Ten patients (36%, 5 had CHT, 5 had no CHT) referred weight loss, febrile episodes or night sweats during the last 6 months; nobody presented fever on the day of the scanning. Fifteen patients (54%, 4 had CHT, 11 had no CHT) had lymphoma localisations outside the brain at PET total body examination. Nobody presented morphological alterations at brain CT examination.

The CHT group underwent to conventional standard-dose chemotherapy (the mean number of cycles was 6 and the mean time elapsed from the treatment was 7 months).

The hydroxydaunorubicin (Adriamycin), bleomycin, vinblastine, dacarbazine (ABVD) chemotherapy was used as a first-line protocol in all the HL patients. The cyclophosphamide, hydroxydaunorubicin and vincristine (Oncovin), prednisone or prednisolone associated with the monoclonal antibody rituximab (R-CHOP) were used as first-line protocol in seven NHL patients (58%).

Second-line treatments were used in five NHL patients (42%), in three cases, in association with rituximab: cyclophosphamide, mitoxantrone (Novantrone), Oncovin, prednisone (CNOP); vincristine, Adriamycin, cyclophosphamide, Oncovin and prednisone (VACOP); and etoposide, Oncovin, cyclophosphamide, hydroxydaunorubicin, (EPOCH). Corticosteroid therapy was used in about 86% (*n* = 12) and immunotherapy in about 71% of patients (*n* = 10). All protocol regimens were considered similar for toxicity and side effects by the oncologists who took care of patients.

All patients underwent to a screening battery, in the same morning before the PET acquisition, to exclude cognitive impairment, anxiety and depression containing the following tests: Mini Mental State Examination (MMSE), Hospital Anxiety and Depression Scale (HADS), Montgomery-Asberg Depression Rating Scale (MADRS), Distress Thermometer (DT).

### PET scanning

In a quiet waiting room, the participants, lying in a supine position, were asked to refrain from moving and instructed ‘to keep their eyes closed, to not engage in any structured mental activity such as counting, rehearsing, etc. and to avoid falling asleep’. They were then blindfolded and ear plugged and received intravenously about 4.5 to 5.5 MBq kg^−1^ of 2-deoxy-2-[^18^ F]fluoro-d-glucose. About 30 min later, PET/CT scan was performed by a Philips Gemini scanner (Philips Medical System, Cleveland, Ohio, USA). The brain scan acquisition time was 20 min. Coronal, sagittal and transverse data sets were reconstructed using a 3D iterative technique (row action maximum likelihood algorithm, RAMLA-3D) and corrected with single scatter simulation. Reconstructed brain images had a dimension of 128 × 128 × 90 voxels (2 × 2 × 2 mm^3^).

PET brain images were preprocessed using SPM8 (http://www.fil.ion.ucl.ac.uk/spm) running on MATLAB 7.5 software (Mathworks, Natick, Massachusetts). All images were nonlinearly and spatially normalized into the Montreal Neurological Institute (MNI) space and smoothed with an isotropic Gaussian kernel with 12 mm full width half maximum. We normalized the count of each voxel to the mean count of a standardized pontine region of interest (ROI). The ROI was a rectangular multislice region (−8 mm < *x* < 8 mm, −32 mm < *y* < −24 mm, −44 mm < *z* < −34 mm, MNI space) sampling 144 voxels on the central pontine region and manually drawn on the PET MNI template using the MRIcron application (https://www.nitrc.org/projects/mricron). The same ROI was employed on each spatial normalized and smoothed brain image to sample the pons mean and then to scale voxel values of each subject individually with the image calculation SPM tool.

### SVM classification analysis

Support vector machine (SVM) is a supervised classification machine-learning algorithm [37] commonly used as neuroimaging MVPA [31,32]. SVM has typically two phases: training and test phase. In the training phase, SVM receives as input a number of cases or instances, described by the values of distinctive features or characteristics, specifying the membership of every case to one of two different groups or categories. For example, we could try to teach to the machine how to classify human gender based on height, hair length and waist to hip ratio features. SVM, in the multidimensional space of the *N* features, searches the best hyperplane **H** (*N*-1 dimensional) separating the classes without error (e.g., females are generally short, long hair and wide hips creatures). Since there could exist more than one solution to this separation problem, SVM searches the only **H** with the maximum margin, which means that the distance or separation of the two classes are maximized by the choice of **H.** This algorithm selects from many possible solutions the optimal one determined by the most informative training examples (called the support vectors), those are the nearest to the margin (borderline cases). This formulation is called hard margin SVM and implies that the problem is well posed, separable and that a solution exists. But there are problems in which some instances could be so misleading, falling quite distant from the representative mean of the group (e.g., a masculine shaved basket girl player), that it does not exist a solution. The soft margin SVM (C-SVM or nu-SVM) introduces a regularization parameter that allows some examples to fall inside the margin and to ignore them in the best choice of **H**. In the proposed example of the shaved girl, that case will be ignored in the choice of **H** and the margin extended to include her. Generally, the value of the parameter should be searched by trials and errors, using some validation criteria to optimize it. After training phase, the algorithm has selected the optimal hyperplane **H** separating the classes that can be described in the following equation:

$$H:wv_{i}+b=0,$$

where **w** are the computed learning weights, *b* is an offset and **v**_*i*_ is the classes features.

In the test phase, another sample of cases is classified using the weights and assigning the membership to a class based on the positive or negative sum of the weighted features (this means to which ‘side’ of the hyperplane the example falls). Therefore, we could verify the accuracy and the generality of the computed solution. Features with a weight near zero tend to have little impact in the classification.

We applied the SVM onto the patient's whole brain PET, where every voxel was considered as a feature and the two classes were CHT or no CHT groups. We chose SVM because it is still effective in high-dimensional spaces, where the number of features is greater than the number of samples (we had 28 cases and thousands of features or voxels). More importantly, it can be taken into account the full spatial pattern of brain activity which is measured simultaneously at many locations and exploiting its inherent multivariate nature which is intrinsically organized in networks.

Analyses were performed with an in-house Matlab-based MVPA toolbox, which adopted LIBSVM, an easy-to-use and efficient library for SVM classification and regression [38]. In particular, we used a nu-SVM classification machine and a grid searching of the best parameter of the machine. The training and test data consist of 28 subjects FDG-PET whole brain data. To test the accuracy of the classification we used *n*-fold validation scheme leaving out two random subjects for the test phase after the training with the remaining 26 subjects, repeating 14 times the procedure. After the best choice of **H**, we had a large number of weights (one for every brain voxels) so we kept only the voxels for which |*w*| > > 0, discarding the other as less important for the classification. We split the weights into two patterns or networks: one of high negative weights and one of high positive weights; these corresponded to the voxels that are important for discrimination between the groups. For the description of these networks, we used the viewer xjView8, an SPM extension that could also output the cortical and Brodmann areas contained within an input brain map in the MNI space ( http://www.alivelearn.net/xjview8).

Then we extracted the mean metabolism from the two patterns of weights and we compared, with two independent sample *t* tests, the values between CHT and no CHT groups. We also compared the mean metabolism with analysis of covariance (ANCOVA) models using potentially confounding as covariate (depression, age and time elapsed from treatment). We calculated the correct classifications of the subjects' membership to CHT or no CHT groups using only the mean metabolic values extracted by the two networks.

We tested the association between clinical, demographic variables and extracted metabolic values with χ^2^ test, two-sample independent *t* test, ANCOVA, Pearson's correlations, Kendall's tau-*b*, using SPSS 13.0; *p* < 0.05 was considered as significant.

## Results

The demographic and clinical characteristics of patients are shown in Table 1. In all patients, except four, HADS scores were under the clinical relevant cut-off of 8 (mildly anxious and depressed and equally distributed among groups). All patients were cognitively spared (MMSE > 26).

**Table 1:** Clinical and demographic characteristics

**Data/subgroups**	**No CHT (*n*** **= 14)**	**CHT (*n*** **= 14)**	***p *Value**^**a**^
Age (years)	52 ± 10	52 ± 10	1.00
Gender (M/F)	11/3	11/3	1.00
Education (years)	14 ± 5	14 ± 5	0.96
Type (HL/NHL)	2/12	2/12	1.00
Age at onset (years)	52 ± 10	51 ± 9	0.96
PET localization outside brain (yes/no)	11/3	4/10	0.01
Lymphoma symptoms (yes/no)	5/9	5/9	1.00
Cycles number (*n*)	-	6 ± 3	-
Post-CHT time (months)	-	7 ± 9	-
First-line treatment^b^ (*n*)	-	9	-
Second-line treatment (*n*)	-	5	-
Immunochemotherapy (*n*)	-	10 (7/3)^c^	-
MMSE/30	28.4 ± 1.3	28.1 ± 1.1	0.63
HADS depression/21	4 ± 3	4 ± 3	0.86
HADS anxiety/21	6 ±3	4 ±3	0.17
MADRS/50	9 ± 6	8 ± 7	0.68
DT/10	3 ±2	2 ±2	0.28

CHT, chemotherapy; F, females; HL, Hodgkin's lymphoma; M, males; NHL, non-Hodgkin's lymphoma. ^a^ Significance *p*, for two independent sample *t* test or *p* Chi-square test. ^b^First-line treatment, ABVD (hydroxydaunorubicin (Adriamycin), bleomycin, vinblastine, dacarbazine) or CHOP (cyclophosphamide, hydroxydaunorubicin, vincristine (Oncovin), prednisone or prednisolone). ^c^Immunochemotherapy, treatment plus immunotherapy (rituximab). Values represent, when not otherwise specified, mean ± standard deviation.

There were no significant differences between groups in level of distress, anxiety, depression, demographic or clinical features (*p* > 0.05).

Patients with and without symptoms, who did not differ in brain metabolism and in clinical and demographic features, were equally distributed among CHT and no CHT groups and had similar presence/absence of PET localizations outside the brain (*p* > 0.05).

### SVM patterns

**Figure 1:**
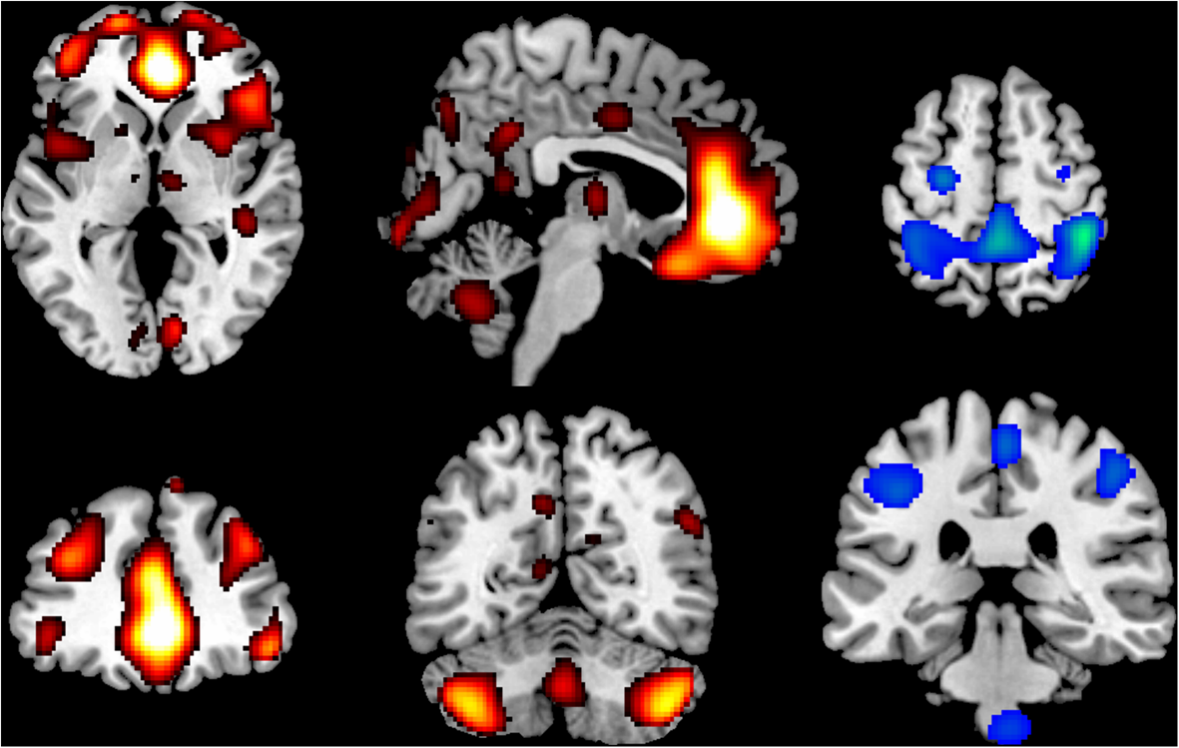
**Patterns of SVM classification analysis.** Weights of SVM classification analysis between no CHT and CHT groups overlaid on canonical MNI (Montreal Neurological Institute) brain template. Images are in neurological convention (left is left). In red the positive weights pattern evidenced a prefrontal cerebellar system (PCS) composed primarily by salience network (SN) and secondarily by central executive network (CEN). In blue the negative weights pattern evidence the dorsal attention network (DAN).

The nu-SVM classification analysis had a good generalization and could discriminate the two groups with an accuracy of 65% (computed by *n*-fold cross validation, greater than chance >57%). The two patterns of more weighting voxels, significant for discrimination, are shown in Figure 1, in red the pattern of high positive weights, in blue the high negative.

The red pattern was symmetrical and included many prefrontal cortices and cerebellar areas: anterior cingulated cortex (ACC, BA 24, 32), medial prefrontal cortex (BA 8, 9, 10) dorsolateral prefrontal cortex (DLPFC, BA 45, 46), orbitofrontal cortex (OFC, BA 11, 47), anterior insula (AI, BA 48), putamen, thalamus, vermis VIII-IX, crus 1, crus 2, lobules VIIB, VIII. It included some additional clusters in the middle cingulated cortex, cuneus, precuneus, calcarine scissure, superior temporal and supramarginal gyrus. The largest part of the pattern, with the highest weights, was contained in front of genu of corpus callosum: ACC and medial prefrontal cortex (BA 10, 32) and in the posterior cerebellum (crus 1 and crus 2). From a network point of view, the pattern was largely overlapping with the SN, but included also parts of the left and right CEN and other additional small clusters. We will refer to this pattern together as the prefrontal cerebellar system (PCS).

The blue pattern was symmetrical and included mostly parietal areas: postcentral gyrus (BA 2, 3), paracentral (BA 5), inferior, superior parietal lobules (BA 7), with additional clusters in precentral cortex (BA 4), particularly the frontal eye field (FEF, BA 6), and in brainstem pontine nuclei. This pattern was virtually indistinguishable from the DAN.

### Classification and groups differences

CHT patients had significantly less positive PET localizations outside brain compared to no CHT patients (*p* = 0.01).

**Figure 2:**
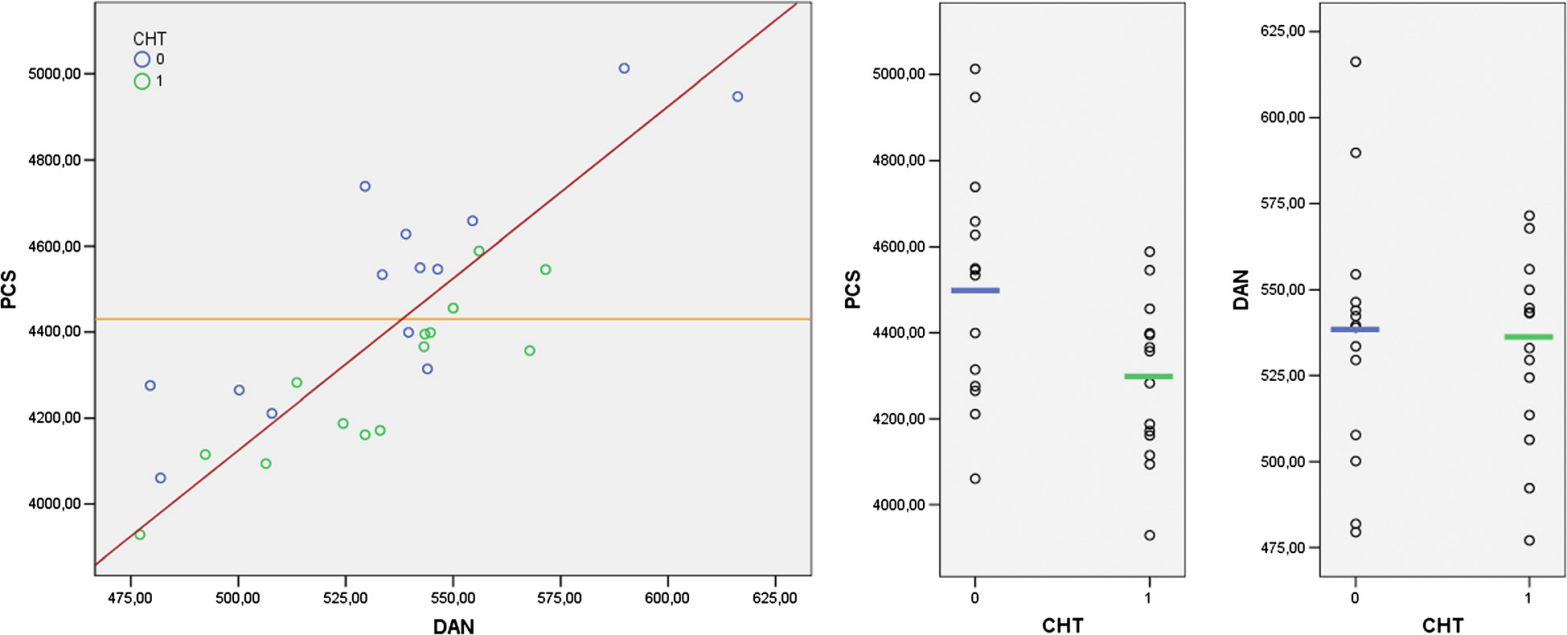
**The mean networks metabolism.** On the left the prefrontal cerebellar system (PCS) and dorsal attention network (DAN) plotted in a scatter dot graph. In blue the no CHT subjects and in green the CHT subjects (arbitrary units). The red line was the best division between CHT and no CHT two groups using both PCS and DAN values. The orange line was the best division between CHT and no CHT using only PCS values. In the middle, the PCS values for the two groups (in green the CHT group = 1, in blue the no CHT group = 0). On the right the DAN, values for the two groups (in green the CHT group = 1, in blue the no CHT group = 0).

The DAN mean metabolism (Figure 2 right) was very similar between groups (two sample *t* test, *p* = 0.77) and largely overlapping (in green CHT, in blue no CHT). The PCS mean metabolism (Figure 2 middle) was significantly (two sample *t* test, *p* = 0.02) lower in the CHT group, but there was a certain amount of subjects with similar values in the two groups. Using the PCS values, searching for the best cut-off to discriminate between CHT (lower than cut-off, under the orange line, Figure 2 left) and no CHT (higher than cut-off, above the orange line, Figure 2 left), we obtained an accuracy of 68%, with a great number of false attribution of patients in no CHT to the CHT group. Using the DAN values in searching for the best cut-off, we obtained a near chance result with only 50% of accuracy. On the contrary, putting together the two values of PCS and DAN with a PCS floating cut-off based on the DAN values (above and below the red line in Figure 2 left) we had an improvement in accuracy to 80%.

The metabolic values of the two networks in all patients were highly positively correlated (PCS-DAN *r* = 0.81, *p* < 0.001) and both exhibited a high inverse correlation with the age of subjects (AGE-DAN *r* = −0.51, *p* = 0.006; AGE-PCS *r* = −0.53, *p* = 0.004). No other clinical or demographic variable was correlated with the metabolic values extracted from the networks.

The ANCOVA models for CHT as independent and PCS mean metabolism as dependent variables were all significant controlling for different covariates: age (*r*^2^ = 0.50, CHT factor *p* = 0.01), depression (*r*^2^ = 0.36, CHT factor *p* = 0.02) or time elapsed from the end of treatment (*r*^2^ = 0.32, CHT factor *p* = 0.04).

## Discussion

The two networks extracted in this work could be compared to those commonly extracted by many groups in the decomposition of fMRI/PET resting state signals, using blind data-driven multivariate techniques as principal component analysis, independent component analysis, subprofile scaling model, fuzzy clustering [35,39,40]. When covariation patterns of activity were extracted from brain resting state, the large-scale spatial networks, intrinsically contained within the mind architecture, were evidenced [20]. These techniques were used in the study of normal subjects to understand brain functioning and in pathological subjects to uncover characteristic pattern useful for diagnosis [41-43].

Some of these networks [23] are more reliable than the others and are detectable in the resting state activity: default mode network, DAN (dorsal attentional network), sensorimotor network, visual networks, auditory network, left and right CEN (central executive networks), salience network (SN). These networks are also present in the activation study when elicited by specific tasks, the similarity of the active networks and resting brain-extracted networks is impressive [23].

Our implementation of MVPA with nu-SVM had the advantage of looking at groups differences considering at the same time the covariation patterns of the voxels in the brain. We found a hypometabolism in a network that we called PCS, composed by SN and to a lesser extent by CEN and OFC. A dysfunction of this network could potentially explain the neurocognitive, behavioural and mood alterations found in some chemotreated cancer survivors [1-3]. Some authors hypothesized that prefrontal dysfunction could be linked to acute depression and posttraumatic stress in those patients that later normalize [44]. However, our patients were not depressed or anxious and were cognitively spared. In our patients, CHT was effective, as demonstrated by the evaluation of total body PET, but nobody suffered severe physical effects. Also controlling for possible confounding effects as depression, time elapsed from CHT or age, the differences between groups in the network evidenced by MVPA was still significant. This result supported the role of CHT as the most probable responsible for the brain hypometabolism in our sample. We also found a spared metabolism in DAN that is important as a reference point for setting the PCS floating cut-off (Figure 2 left).

The meaning of these neural patterns could be inferred by meta-analytic analyses looking at the functional interpretations of brain networks in resting state [22].

In particular, the PCS included the cingulo-insular SN, an aspect of the attention and monitoring of the priority of stimuli processing and fronto-parietal CEN linked to working memory, planning, execution and action sequencing and the orbitofrontal areas related to reward, motivation and goal driven behaviour [22]. The DAN has constantly been associated with attention orientation, especially eyes orientation, and focusing into space, visuospatial integration, coordination and execution [22]. The presence in the DAN network of sensory-motor cortex and midbrain was extremely interesting and could reflect a link with coordination/execution, arousal and attentional processes.

All this functions are very flexible, involved in many tasks and are all often grouped under the name of frontal or executive functions; therefore, it is plausible to have seen them cluster together. The DAN performs less cognitive tasks, but highly interconnected with the aforementioned functions. In fact, focusing and orienting toward interesting things and planning/execution of goal-oriented actions are a top-down mechanism mirror of the bottom-up attention saliency: This was reflected in the DAN and PCS as very high positive correlation that we found in our sample. Both DAN and PCS are substrate of flexible, attention-based rapid processing skills that depend heavily on age and have a marked trend to decline with it [45]. In our sample, both DAN and PCS metabolism exhibited a high inverse correlation only with the age of subjects, and this is consistent with the functions that we attributed to these networks. We therefore could summarize this finding as a general inclination to have a global level of functioning that decreased in subjects with ageing. However, there is an important, although slight, difference in the two groups: The CHT seemed to dip only into the PCS, widely sparing the DAN metabolism. Age obviously represents a good coarse indicator of general frontal functioning, but different subjects of the same age could have different global frontal functioning due to other factors (genes, experiences, learning, epigenetic and environment). In this case, the best thing to study brain metabolism is to have a good indicator that could act as a set point as the DAN metabolism, unaffected by CHT and so equal to the pre-treatment level. This indicator definitively improved our capacity to discriminate between CHT and no CHT subjects (from 50% to 80%). We demonstrated a specific effect of the CHT (no other variable are significant in the comparison between groups, see Table 1 and ANCOVA analyses) on a specific network (PCS). CHT had a small effect, which is hard to detect or put in the right context with traditional univariate analyses [10-13] or for behavioural comparison (no detectable differences between CHT and no CHT in any cognitive or psychological data).

Our findings could be juxtaposed with the recent work of Bruno and co-workers [46] that used the graph theoretical analysis to examine the connectome in chemotherapy-treated breast cancer survivors. They found a disrupted regional network in frontal, striatal and temporal areas, which is very similar to the PCS hypometabolic network.

### Limitation

The limitation of the study was the presence in the sample of immunotherapy and the use of steroid at different doses or second-line treatments that do not allow us to distinguish if some chemotherapy drugs or combination could have more impact than the other. However, this limitation became less important, as we used a technique with increased sensitivity and we focused on a multivariate networks analysis prospective.

### Future directions

We could hypothesize a future clinical use of the networks’ perspective in extracting PCS and DAN metabolic values from standard ROIs to identify patients at risk, using only one FDG-PET scan, and possibly to treat before clinical manifestation of cognitive fog or cancer fatigue occurs. This work should be replicated in greater numbers and with longitudinal design to demonstrate the clinical applicability, and hypometabolism should be put in relation to the development of clinical symptoms.

We could investigate possible mechanisms of neurotoxicity (e.g., proinflammatory cytokines, toxicity via brain blood barrier transporters and oxidative stress) via comparison of networks metabolism in groups stratified by genes' polymorphism (e.g., multi-drug resistant protein or MRP, interleukin or IL, tumour necrosis factor or TNF, apolipoprotein E or APOE, brain-derived neutrotrophic factor or BDNF).

## Conclusion

The emerging concept consisted in the importance of the investigation of brain intrinsic networks, using multivariate statistical analyses, in cognitive chemotherapy, induced changes.

## Competing interests

The authors declare that they have no competing interests.

## Authors' contributions

FD and BB have made substantial contributions to conception and design. FD, PC, BB, GG, LP, GC and GB are involved in the acquisition of data. FD, TC, BB, FC and PM are responsible for the analysis and interpretation of data. All authors have been involved in drafting the manuscript or revising it critically for the important intellectual content. All authors read and approved the final manuscript.
